# Development of a New Fleet Disease and Injury Surveillance Capability Using ESSENCE

**Published:** 2025-02-20

**Authors:** Wendi S. Bowman, Sasha A. McGee, Lisa A. Pearse, Courtney Coker, Jamaal A. Russell, Asha J. Riegodedios

**Affiliations:** 1 Battelle Memorial Institute, supporting U.S. Navy and Marine Corps Force Health Protection Command, Portsmouth, VA; 2 Integrated Biosurveillance Branch, Armed Forces Health Surveillance Division, Public Health Directorate, Defense Health Agency, Silver Spring, MD; 3 Navy and Marine Corps Force Health Protection Command, Portsmouth

## Abstract

Historically, disease and illness (D&I) surveillance on U.S. Navy vessels relied on weekly data updates and required manual data processing. Established surveillance approaches for fixed military hospitals and clinics were not designed to be applied to the highly mobile populations aboard ships. This paper describes the development of a new surveillance capability through utilization of the Electronic Surveillance System for the Early Notification of Community-based Epidemics (ESSENCE). The pilot program successfully instituted a near real-time D&I surveillance system defined for shipboard operations. Following initial data and system assessment, an operational surveillance strategy was developed and implemented at the Navy’s 4 regional Navy Environmental and Preventive Medicine Units responsible for global fleet assets. Despite early implementation challenges, preventive medicine users reported that the fleet ESSENCE system was effective in identifying potential outbreaks, with sufficient efficiency for daily surveillance.

**What are the new findings?:**

This new capability using in-theater data in ESSENCE enables unprecedented, near real-time D&I surveillance for the U.S. Navy fleet. While currently targeting gastrointestinal and respiratory illness trends, the infrastructure has flexibility to add new modules in response to fleet and preventive medicine requirements.

**What is the impact on readiness and force health protection?:**

High quality D&I surveillance of operational forces by Navy preventive medicine assets accelerates technical support and response to outbreaks and other public health threats. Rapid implementation of appropriate control measures is the key to minimizing the effect of these events on both the force and the mission.

## 1 BACKGROUND

Force protection against public health threats depends on timely, accurate public health surveillance data. A robust and flexible disease and illness (D&I) surveillance system is imperative for the U.S. Department of the Navy, due to its highly mobile population with frequent missions to isolated and resource-limited locations around the globe, confined living conditions aboard ships, and the dynamic nature of diseases.

Historically, D&I surveillance involved labor-intensive, manual methods that produced weeks-long delays in situational awareness.^1,2,3,4,5^ U.S. Navy vessels have since adopted electronic health record (EHR) capabilities, allowing more time-efficient D&I surveillance methods. Shipboard medical visits are entered into Armed Forces Health Longitudinal Technology Application-Theater (AHLTA-T) or Shipboard Automated Medical System (SAMS), employing a ‘store and forward’ model designed for low communication environments; data are stored until internet connectivity is available, at which time they are transmitted to a central data repository, the Theater Medical Data Store (TMDS). With the recent addition of TMDS data into the Department of Defense (DOD)’s Electronic Surveillance System for the Early Notification of Community-based Epidemics (ESSENCE), the Navy and Marine Corps Force Health Protection Command (NMCFHPC) proposed an initiative to advance an automated D&I surveillance capability.

Millions of outpatient medical encounter records and laboratory results are systematically queried using ESSENCE, to detect health events of potential public health significance and support public health investigations.^6^ Since 2003, ESSENCE began supporting force health protection by collecting near real-time health surveillance data on U.S. military health system beneficiaries from on-base, fixed location military hospitals and clinics. Beginning in 2017, the Armed Forces Health Surveillance Division (AFHSD) Integrated Biosurveillance Branch (IB) worked to acquire mobile, forward-operating clinical data from the TMDS, structured those data for ESSENCE integration, and collaborated with security experts to mitigate potential risks associated with data access. By October 2022, TMDS data became available to selected ESSENCE users for evaluation and pilot testing and, since June 28, 2024, have been ingested into ESSENCE in batches every 12 hours. This integration of TMDS data with ESSENCE provided the NMCFHPC with an opportunity to improve maritime situational awareness.

This report details the steps taken to develop a timely, accurate, and comprehensive Navy fleet D&I surveillance capability, along with the successes and challenges that will guide further refinement and expansion of this tool.

## 2 METHODS

From October 2022 until June 2023, AFHSD-IB and NMCFHPC worked together to develop and test the initial surveillance capability. The implementation plan 1) assessed TMDS data quality and the utility of available ESSENCE analytic tools, 2) developed an initial shipboard surveillance capability for regional surveillance, 3) recommend and implemented ESSENCE system improvements, and 4) tested and evaluated the capability.

**Data Assessment**

An initial assessment of ESSENCE TMDS data in January 2023 demonstrated a total of 246 data fields, including many necessary for operational health surveillance, such as patient and reporting unit, demographic fields, clinical notes and vital statistics, laboratory and pharmacy data, discharge diagnosis codes, chief complaints, and D&I category fields. While many fields were sufficiently complete for both surveillance and disease threat characterization, they were often difficult to query due to unstructured formats (i.e., use of free text). The completeness of ship data was evaluated using the Navy Vessel Register.^7^ The list of expected ships (excluding inactive ships, those in Navy Sealift Command, and forward medical units not identified as ships) were compared to ships with data recorded in ESSENCE at least once from January 2022 through December 2023.

From January through June 2023, over 75,000 health care encounters on U.S. Navy fleet vessels were captured in ESSENCE. Approximately 81% of expected ships had encounters documented. The distribution of health encounters, by ship size, is shown in **Table 1**.

Data timeliness was assessed based on the difference between the date of the health care encounter and when the data were uploaded into ESSENCE, for those ships with data in ESSENCE (**Table 2**). An ESSENCE upload date signifies the most recent date a record is updated rather than the date the record was first received, so observed timeliness in **Table 2** may overestimate the true interval. Within 10 days, 78% of clinical encounters were visible in ESSENCE. Encounter data from smaller ships were not as timely as data captured from larger ships.

**System Assessment**

NMCFHPC’s qualitative review of ESSENCE’s functionality and capability revealed several issues that required resolution with the AFHSD-IB ESSENCE team. In some cases, the ESSENCE developers modified the system’s functionality to address limitations. Several modifications were implemented to improve user experience and better meet surveillance needs (**Table 3**). Other issues were addressed through ESSENCE queries designed to minimize data quality limitations.

**Shipboard Surveillance Pilot**

**Figure 1:**
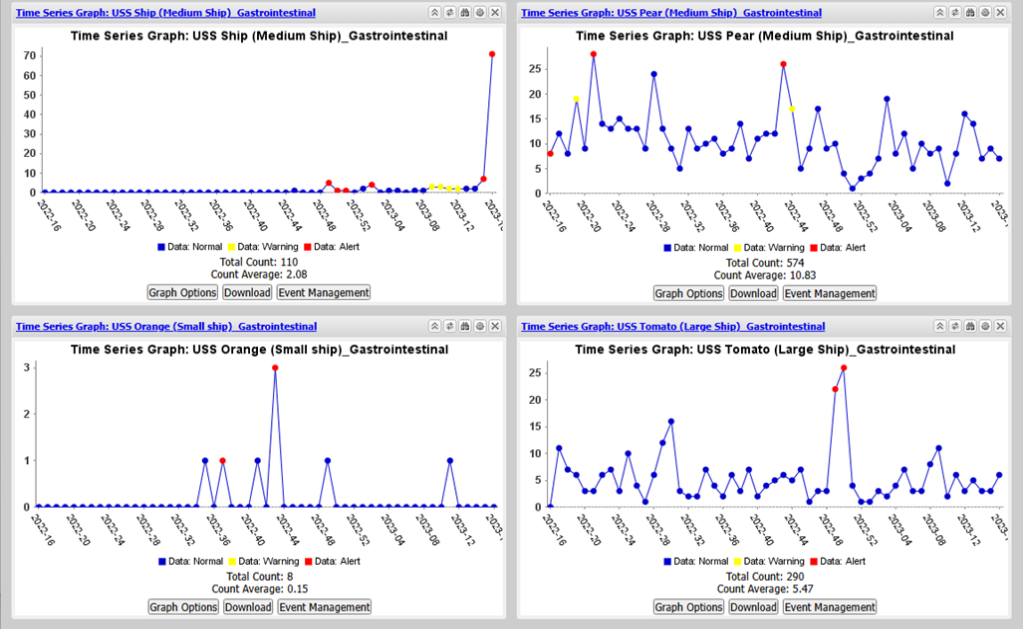
Time Series Graphs^a^ for Gastrointestinal Diseases Reported from Individual U.S. Navy Ships ^a^Within each dashboard, time series graphs represent weekly trends of a single ship's encounters for a specific syndrome category. Note: ESSENCE alerting algorithms test for unusually high counts compared to what is expected based on the baseline, preceding time period. A yellow alert indicates that the statistical significance (p-value) is less than 0.05, while a red alert indicates that the statistical significance is less than 0.01.

NMCFHPC’s fleet surveillance methodology for the pilot program involved the creation of dashboards to visually display time series graphs of the query results. A series of graphs were initially generated to determine the best way to aggregate data for ships (e.g., as a function of ship size, geography, mission relevancy, syndrome category) to facilitate efficient data review. Displaying data for a single ship in each time series graph was found to be optimal for ease of data review and interpretation (**Figure 1**).

Three outcomes of interest were selected to be displayed on dashboards as time series graphs: all daily health care encounters for the past 3 months, weekly gastrointestinal illness encounters for the past year, and weekly respiratory illness encounters for the past year. Ships were divided into 4 geographic areas, representing each of the Navy’s 4 regional Navy Environmental and Preventive Medicine Units (NEPMUs), based on home port as indicated in the Naval Vessel Register.^7^ Time series graphs for all ships associated with a specific NEPMU (range: 16-73 ships) and specific outcome were displayed on a single dashboard. In the end, over 600 time series graphs were developed to form the final set of 12 total dashboards (with 3 outcomes per NEPMU).

Fleet surveillance was initiated for all 4 NEPMUs from April through June 2023, following individual training and distribution of a companion training guide. Each NEPMU had 1 to 3 users (either environmental health officers, preventive medicine physicians, or preventive medicine technicians) who were tasked with reviewing the dashboards (**Figure 1**) at least twice per week to identify trends that indicated a potential public health concern. When unusual trends were observed, NEPMUs viewed a listing of individual encounter data (clinical notes, demographics, discharge diagnosis, lab results) for a specific date to facilitate their initial public health threat assessment. Findings suggesting a potential outbreak triggered communication between NEPMU and the ship for support.

**Figure 2:**
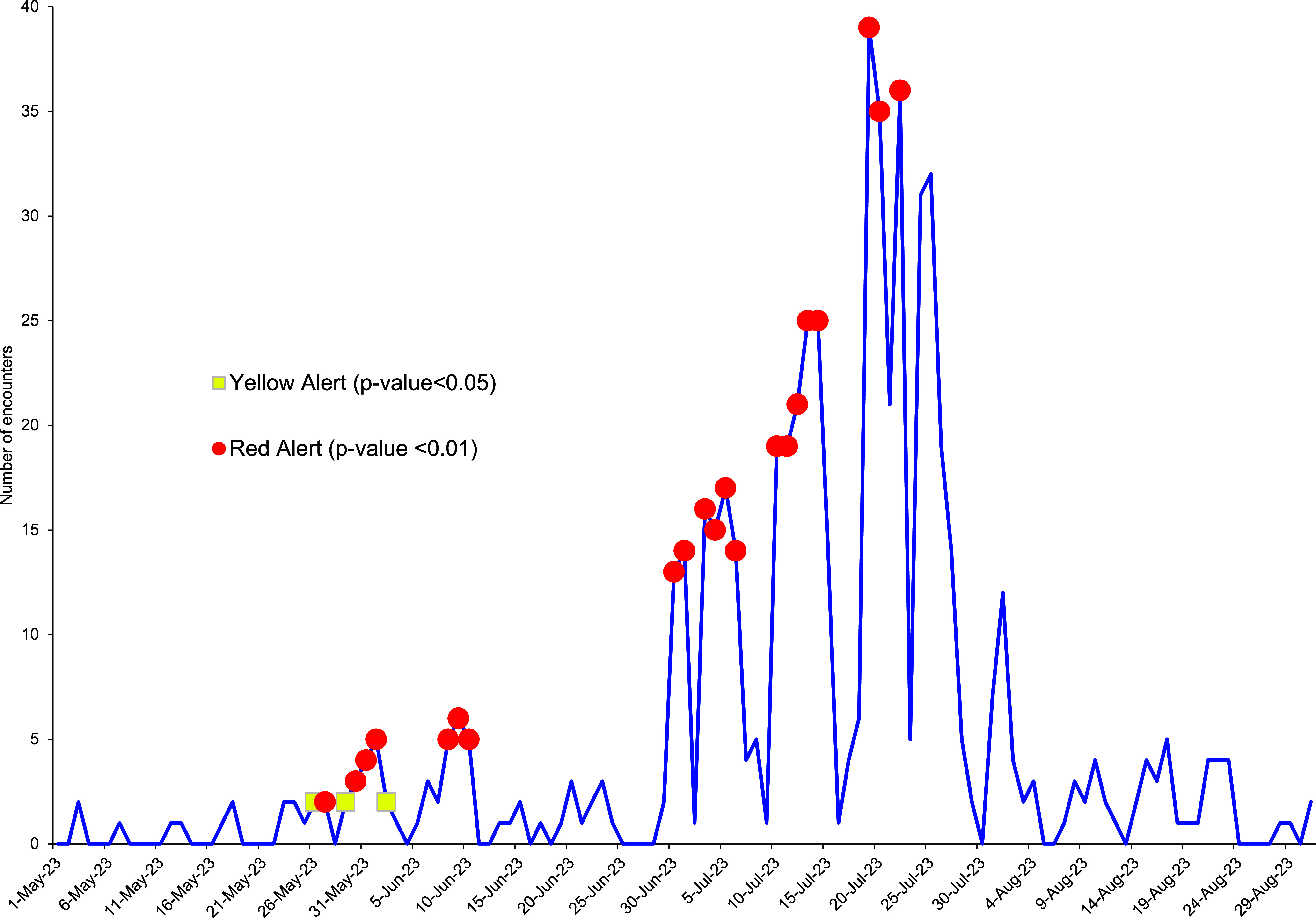
Gastrointestinal Health Encounters Onboard a U.S. Navy Ship Experiencing an Outbreak, May 2023-August 2023

During the pilot program, 1 NEPMU began closely monitoring a large ship with an apparent gastrointestinal outbreak. Before initiating contact with the ship, a risk assessment was completed within minutes, based solely on the ESSENCE data details. Analysis revealed that most patients had similar symptoms, and before their illness, many patients reported consuming street food during a recent port visit. Norovirus was laboratory confirmed as the etiologic agent. Details were confirmed upon direct communication with the fleet. The ESSENCE gastrointestinal illness dashboard continued to be used for ongoing monitoring of control measure effectiveness during the outbreak, which took more than 3 weeks to resolve (**Figure 2**).

Three months after the pilot program was initiated, user responses on the utility of the ESSENCE shipboard dashboards, as an integrated part of routine surveillance at the NEPMU, were collected via electronic survey, administered with Microsoft 365 Forms. Virtual user forums served as a mechanism for gathering additional details on strengths and limitations, developing potential solutions to those limitations, and informing a plan to expand the capability throughout the fleet public health community.

Responses indicated that each NEPMU had at least 1 intermediate or advanced user with prior ESSENCE experience. The frequency of dashboard review varied depending upon ship distribution within a regional area. The NEPMU with the fewest ships reported that dashboard review once a week was sufficient, due to other available surveillance methods; NEPMUs with more ships reported reviewing their dashboards daily. NEPMUs reported being able to easily identify concerning trends using the dashboards within 15-30 minutes, with additional time needed when a review of underlying data was necessary. Users also noted timely data updates for many ships within ESSENCE, particularly ships with larger populations. Notable challenges included reports of the system being slow at times, and low numbers of encounters that complicated trend detection and quick risk assessments. Users also reported that data interpretation was complicated by a lack of understanding of various EHR data entry challenges aboard ships, such as software technical issues, paper record use, and intermittent electronic communication access.

## 4 DISCUSSION

This report recounts a major advancement in timely and reliable public health surveillance for ships, made possible through use of ESSENCE TMDS data. Surveillance methodology using ESSENCE for on-base military hospitals and clinics could not be applied to fleet surveillance due to differences in both data structure and populations served (i.e., smaller, healthier, closed populations aboard ships).^8^ This pilot program developed, within 3 months, a new capability to monitor mobile populations ranging from 50 to 5,000 people that addressed their complexities and unique challenges.

In the past, D&I surveillance involved collecting and compiling reports from individual ships, a time-intensive multi-step process, but now data are automatically collected and available every 12 hours, a major advancement. This new capability supports expeditious and efficient data review, facilitates communication between the fleet and preventive medicine experts, and contributes to disease outbreak identification and containment.

Initial data assessments for this pilot program revealed remarkably higher levels of completeness and timeliness compared to legacy D&I surveillance strategies.^4,5,9^ Nearly three-quarters of encounters for ships (with all sizes combined) were visible within 7 days, a notable improvement over the weeks-long delays with earlier methods. These gains in data timeliness and completeness were achieved without requiring additional time or effort from a ship’s medical staff. Nonetheless, the delay between the health care encounter date and the ESSENCE upload date is a potential limitation that may require further study to improve this surveillance capability.

Several challenges had to be overcome for this pilot program’s success. Lack of standardized discharge diagnostic code usage was problematic, likely due to lack of synchronization of updates to shipboard information technology. For ships still using International Classification of Diseases, 9th Revision, Clinical Modification codes, queries were developed using chief complaint text. The field containing the ship name was unstructured (i.e., utilized free text) and names were not entered using a single standardized naming convention, presenting another major barrier. Hundreds of queries had to be developed and refined to obtain reliable results for ship-specific data. The final set of queries were complex, as a result of accounting for various naming patterns observed in the data. Periodic data review and revisions will be necessary to ensure queries continue to reliably capture ship data as expected. Ongoing, collaborative engagement between military surveillance experts (AFHSD-IB and NMCFHPC), the ESSENCE developers, and theater data owners was essential for the success of this pilot program.

Two major challenges remain. The first challenge is the need to develop more efficient methods of surveilling shipboard populations with low numbers of health care encounters. Medical departments on smaller ships may only see 5-15 patients a week, making the determination of daily trends for specific outcomes (e.g., gastrointestinal illness, respiratory illness) difficult. The surveillance of all health care encounters, instead of individual syndromes, was evaluated as a solution but was further complicated by large numbers of periodic administrative encounters that interfered with the detection of potential public health threats. The second challenge involves intermittent data gaps in ship time series graphs, which can interfere with data trend interpretation. Anecdotal evidence suggests that these gaps are related to routine shipboard operations (e.g., maintenance, pulling into port). Geographic-specific operations or EHR system technical limitations may also lead to temporary use of paper medical records. More study is needed to fully assess these occurrences and develop approaches to improve the reliability of fleet surveillance.

This new capability provides an extraordinary opportunity to expand and improve operational fleet D&I surveillance. The methods and framework developed by this pilot program can be further adapted and expanded for surveillance of other health events of interest, such as injuries and mental illnesses. Additionally, the availability of near real-time data that are accessible by public health responders is ideal not only for threat detection, but reviewing and pursuing data quality improvements. Although mechanisms may differ, expansion efforts are being pursued. ESSENCE TMDS data were used for surveillance during a military exercise, Exercise Talisman Sabre 2023, and provided effective, timely public health information beyond outbreak-specific surveillance. Near real-time D&I surveillance promotes enhanced situational awareness at regional commands as well as headquarters, facilitating development of operational plans that can mitigate potential public health threats as early as possible.ng, and innovative tools to cause code efficiently and accurately. Ultimately, knowledge of causes is a foundation for the reduction of the burden of injuries on the military medical system and sustainment of military medical readiness.

## Figures and Tables

**Table 1:** Percentage of Ships with Health Care Encounter Data in ESSENCE by Ship Size, January-June 2023

Ship category (Population Size)	% of ships
Large ships (>=5000)	91.0
Medium ships (1000-3000)	100.0
Small ships (<=500)	78.0

**Table 2:** Percentage of Health Encounter Records Received by Time Interval and Ship Size, January-June 2023

Ship category (Population Size)	<=3 days (%)	<=7 days (%)	<=10 days (%)	<=14 days (%)	<=21 days (%)	<=28 days (%)
Large ships (>=5000)	67.2	78.4	83.3	87.6	91.3	93.1
Medium ships (1000-3000)	57.3	68.9	72.7	75.8	80.6	85.2
Small ships (<=500)	50.4	63.0	68.2	74.1	80.8	85.5
Total	62.1	73.6	78.4	82.7	87.3	90.2

^a^Time intervals were calculated as number of days between encounter date and ESSENCE upload date.

**Table 3:** Observations, Findings, and Associated Actions for Development of Fleet Surveillance Capability Using ESSENCE TMDS Data

Observations and Findings	Associated Actions
Multiple records (rows) per encounter for multiple lab test results for same patient, resulting in inflated health encounter counts	Laboratory data for a single encounter were concatenated ("flattened") into a single row
Three primary D&I fields could be used to develop queries	D&I field based on ICD-10-CM code was selected to develop queries, given high level of completeness and alignment with clinical trials
Some ships (26%) used outdated ICD-9-COM codes for discharge diagnosis categorization	D&I field based on chief complaints was selected to develop queries for ships using ICD-9-CM codes
Intermittent data gaps for time series graphs of shipboard health care encounters complicated data interpretation	Time series graphs with all health care encounters were included in surveillance dashboards for review, in addition to specific syndromes, to enable monitoring of incoming data consistency
Lack of standard naming convention for text field containing ship name	Queries were developed to account for name variations
Inability to directly query data field containing ship name	ESSENCE query options were updated to enable direct free text queries of the field
Lack of general query that captured all respiratory illness, a necessity for small population surveillance	Built-in query was developed to capture a broad range of acute respiratory illnesses
More than 200 data fields possible for a single health care encounter, complicating record reviews	ESSENCE was updated so data fields were rearranged in order of epidemiological importance, and irrelevant fields were hidden

Abbreviations: ESSENCE, Electronic Surveillance System for the Early Notification of Community-based Epidemics; TMDS, Theater Medical Data Store; D&I, disease and injury; ICD-10-CM, International Classification of Diseases, 10th Revision, Clinical Modification; ICD-9-CM, International Classification of Diseases, 9th Revision, Clinical Modification.

## AUTHOR AFFILIATIONS

Battelle Memorial Institute, supporting U.S. Navy and Marine Corps Force Health Protection Command, Portsmouth, VA: Ms. Bowman; Integrated Biosurveillance Branch, Armed Forces Health Surveillance Division, Public Health Directorate, Defense Health Agency, Silver Spring, MD: Dr. McGee, Dr. Russell; Navy and Marine Corps Force Health Protection Command, Portsmouth: Ms. Coker, Dr. Pearse, Ms. Riegodedios

## DISCLAIMER

The views expressed in this article are those of the authors and do not necessarily reflect official policy nor position of the Department of Defense, Defense Health Agency, Department of the Navy, or the U.S. Government. Opinions, interpretations, conclusions, and recommendations are those of the authors and are not necessarily endorsed by the U.S. Navy. Several of the authors are U.S. Government employees. This work was prepared as part of official duties. Title 17 U.S.C. §105 provides that Copyright protection under this title is not available for any work of the United States Government. Title 17 U.S.C. §101 defines a U.S. Government work as a work prepared by a military service member or employee of the U.S. Government as part of that person’s official duties. This material is based upon work supported by the DOD Information Analysis Center Program Management Office (DoD IAC PMO) and sponsored by the Defense Technical Information Center (DTIC) and the Navy and Marine Corps Force Health Protection Command (NMCFHPC) under contract FA807518D0005-FA807523F0016.

## References

[1] Navy and Marine Corps Public Health Center Navy and Marine Corps Public Health Center Technical Manual NMCPHC-TM 6220.12: Medical Surveillance and Reporting..

[2] Shaw E, Hermansen L, Pugh W Disease and Non-Battle Injuries Among Navy and Marine Corps Personnel During Operation Desert Shield/Desert Storm..

[3] Pugh WM A Strategy for Computing Disease and Non-Battle Injury Rates..

[4] Kauvar DS, Gurney J (2020). Exploring nonbattle injury in the deployed military environment using the Department of Defense trauma registry.. Mil Med..

[5] Bohnker BK, Sherman SS, McGinnis JA (2003). Disease and nonbattle injury patterns: afloat data from the U.S. Fifth Fleet (2000-2001).. Mil Med..

[6] Burkom H, Loschen W, Wojcik R (2021). Electronic surveillance system for the Early Notification of Community-Based Epidemics (ESSENCE): overview, components, and public health applications.. JMIR Public Health Surveill..

[7] Naval Sea Systems Command Navy Vessel Register..

[8] Meadows SO, Engel CC, Collins RL 2018 Department of Defense Health Related Behaviors Survey (HRBS): results for the active component..

[9] Hauret KG, Pacha L, Taylor BJ, Jones BH (2016). Surveillance of Disease and Nonbattle Injuries During US Army Operations in Afghanistan and Iraq.. US Army Med Dep J..

